# Development of acquired resistance to lapatinib may sensitise HER2-positive breast cancer cells to apoptosis induction by obatoclax and TRAIL

**DOI:** 10.1186/s12885-018-4852-1

**Published:** 2018-10-11

**Authors:** Alex J Eustace, Neil T Conlon, Martina S J McDermott, Brigid C Browne, Patrick O’Leary, Frankie A Holmes, Virginia Espina, Lance A Liotta, Joyce O’Shaughnessy, Clair Gallagher, Lorraine O’Driscoll, Sweta Rani, Stephen F Madden, Neil A O’Brien, Charles Ginther, Dennis Slamon, Naomi Walsh, William M Gallagher, Radoslaw Zagozdzon, William R Watson, Norma O’Donovan, John Crown

**Affiliations:** 1 Molecular Therapeutics for Cancer Ireland, National Institute for Cellular Biotechnology, Dublin City University, Dublin, Ireland; 2 UCD School of Biomolecular and Biomedical Science, Conway Institute of Biomolecular and Biomedical Research, University College Dublin, Dublin, Ireland; 3 Texas Oncology-Memorial Hermann Memorial City, US Oncology Research, 925 Gessner Road #550, Houston, TX 77024-2546 USA; 4 George Mason University, Manassas, VA USA; 5 Baylor-Sammons Cancer Center, Texas Oncology, US Oncology, Dallas, TX USA; 6 School of Pharmacy & Pharmaceutical Sciences, and Trinity Biomedical Sciences Institute, Trinity College Dublin, Dublin, Ireland; 7 Data Science Centre, Royal College of Surgeons in Ireland, Dublin, Ireland; 8 Division of Hematology-Oncology, Department of Medicine, David Geffen School of Medicine, University of California at Los Angeles, California, Los Angeles USA; 9 Department of Clinical Immunology, Transplantation Institute, Medical University of Warsaw, Nowogrodzka, 59 Warsaw, Poland; 10 Department of Oncology, St. Vincent’s University Hospital, Dublin, Ireland

**Keywords:** ErbB2, MCL-1, AKT, FOXO3a, cFLIP

## Abstract

**Background:**

Lapatinib has clinical efficacy in the treatment of trastuzumab-refractory HER2-positive breast cancer. However, a significant proportion of patients develop progressive disease due to acquired resistance to the drug. Induction of apoptotic cell death is a key mechanism of action of lapatinib in HER2-positive breast cancer cells.

**Methods:**

We examined alterations in regulation of the intrinsic and extrinsic apoptosis pathways in cell line models of acquired lapatinib resistance both in vitro and in patient samples from the NCT01485926 clinical trial, and investigated potential strategies to exploit alterations in apoptosis signalling to overcome lapatinib resistance in HER2-positive breast cancer.

**Results:**

In this study, we examined two cell lines models of acquired lapatinib resistance (SKBR3-L and HCC1954-L) and showed that lapatinib does not induce apoptosis in these cells. We identified alterations in members of the BCL-2 family of proteins, in particular MCL-1 and BAX, which may play a role in resistance to lapatinib. We tested the therapeutic inhibitor obatoclax, which targets MCL-1. Both SKBR3-L and HCC1954-L cells showed greater sensitivity to obatoclax-induced apoptosis than parental cells. Interestingly, we also found that the development of acquired resistance to lapatinib resulted in acquired sensitivity to TRAIL in SKBR3-L cells. Sensitivity to TRAIL in the SKBR3-L cells was associated with reduced phosphorylation of AKT, increased expression of FOXO3a and decreased expression of c-FLIP. In SKBR3-L cells, TRAIL treatment caused activation of caspase 8, caspase 9 and caspase 3/7. In a second resistant model, HCC1954-L cells, p-AKT levels were not decreased and these cells did not show enhanced sensitivity to TRAIL. Furthermore, combining obatoclax with TRAIL improved response in SKBR3-L cells but not in HCC1954-L cells.

**Conclusions:**

Our findings highlight the possibility of targeting altered apoptotic signalling to overcome acquired lapatinib resistance, and identify potential novel treatment strategies, with potential biomarkers, for HER2-positive breast cancer that is resistant to HER2 targeted therapies.

**Electronic supplementary material:**

The online version of this article (10.1186/s12885-018-4852-1) contains supplementary material, which is available to authorized users.

## Background

Lapatinib, the dual kinase inhibitor which targets HER2 and EGFR, has anti-tumour activity against HER2-positive breast cancer cells, including trastuzumab resistant cells [1]. The mechanism of action of lapatinib differs from trastuzumab, as lapatinib can induce apoptotic cell death in HER2-positive breast cancer cells whereas trastuzumab does not, at least in vitro [2, 3]. Alterations in proteins regulating both the intrinsic and extrinsic pathways of apoptosis have been implicated in lapatinib resistance in preclinical studies, including members of the inhibitor of apoptosis protein (IAP) family [4, 5], members of the B-cell CLL/lymphoma 2 (BCL-2) family [6] and TNF-related apoptotic inducing ligand (TRAIL) receptors [6].

BCL-2 family members regulate the intrinsic pathway of apoptosis. The pro-survival (e.g. myeloid cell leukemia sequence 1 (BCL2-related) (MCL-1), BCL-2 and BCL2-like 1 (BCL-X_L_)) and pro-apoptotic (e.g. BCL2-associated X protein (BAX), BCL2-antagonist/killer 1 (BAK) and BCL2-associated agonist of cell death (BAD)) members of the BCL-2 family interact on the surface of the mitochondria, controlling the release of cytochrome-c and the induction of the caspase cascade. In colon cancer cells, extended exposure to lapatinib resulted in increased expression of MCL-1, decreased BAX expression, and inactivation of BAK. Furthermore, knockdown of MCL-1 enhanced the effect of lapatinib whilst knockdown of BAK resulted in the inhibition of apoptosis in the colon cancer cells [6, 7].

Obatoclax (GX15–070), is a BCL-2 homology doman 3 (BH3) mimetic which binds to a broad spectrum of BCL-2 family members including BCL-2, BCL-X_L_ and MCL-1. Obatoclax antagonises MCL-1 by inhibiting the formation of MCL-1/BAK dimers on the mitochondrial membrane [8], and promotes apoptosis. Obatoclax enhanced sensitivity to lapatinib in colon and breast cancer cell lines [6, 7]. Mitchell et al. [9] showed that either knockout of MCL-2 and BCL-X_L_ or treatment with obatoclax enhanced response to lapatinib in HER2-positive breast cancer cells.

In the extrinsic pathway of apoptosis, TRAIL ligand acts by binding to the TRAIL death receptors (TRAIL-1 and TRAIL-2), and initiates apoptosis by inducing the formation of the death inducing signalling complex (DISC). HER2 targeting by short-term treatment with trastuzumab increased expression of TRAIL receptors in SKBR3 cells and increased sensitivity to TRAIL-induced apoptosis [10]. Dolloff et al. [11] showed that pre-treatment of a panel of TRAIL resistant colon cancer cell lines with lapatinib sensitized the cells to TRAIL, resulting in caspase activation and apoptotic cell death.

In this study, we examined alterations in regulation of the intrinsic and extrinsic apoptosis pathways in cell line models of acquired lapatinib resistance, and investigated potential strategies to exploit alterations in apoptosis signalling to overcome lapatinib resistance in HER2-positive breast cancer.

## Methods

### Cell culture

SKBR3 and HCC1954 were obtained from the American Tissue Culture Collection (ATCC). Cell lines were maintained at 37 °C with 5% CO_2_ in RPMI supplemented with 10% FCS. SKBR3-L and HCC1954-L cells were established by twice weekly treatments with 250 nM and 1250 nM lapatinib, respectively, for 6 months which resulted in the cell lines achieving IC_50_ values to lapatinib of 6.5 ± 0.4 μM or 2.67 ± 0.08 μM respectively [12]. All cell lines were fingerprinted by Source Bioscience™ (Additional file 6: Supplementary materials and methods 1) and underwent regular mycoplasma testing both before and after the study. Stock solutions of lapatinib (10 mM) (GlaxoSmithKline), and obatoclax (10 mM) (Selleck Chemicals) were prepared in dimethyl sulfoxide (Sigma-Aldrich). TRAIL (Peprotech) (100 mg/ml) was prepared in UltraPure Water.

### Proliferation assay

Proliferation was measured using an acid phosphatase assay. For dose response assays, SKBR3-Par and SKBR3-L cells were seeded at 3 × 10^3^ cells/well in a 96-well plate, whilst HCC1954-Par and HCC1954-L were seeded at 2 × 10^3^ cells/well. Plates were incubated overnight at 37 °C followed by addition of drug at the appropriate concentrations. Cells were incubated for a further 5 days. For combination assays of LY294002 and TRAIL, SKBR3-Par cells were seeded at 6 × 10^3^ cells/well. Following overnight incubation drugs were added at the appropriate concentrations. LY294002 inhibitor was added for 6 h prior to the addition of TRAIL. Plates were then incubated at 37 °C for 72 h. The wells were washed once with PBS, then phosphatase substrate (10 mM paranitrophenyl phosphate (Sigma Aldrich) in 0.1 M sodium acetate buffer with 0.1% Triton X (Sigma Aldrich)) was added to each well and incubated at 37 °C for 1 h. 50 μl of 1 M NaOH was added and the absorbance was read at 405 nM (reference - 620 nM).

### Microarray analysis

Microarray analyses were performed for the SKBR3-Par and SKBR3-L cell lines. Briefly, cells were grown to log phase and RNA was extracted using the RNeasy Kit (Qiagen). The purified RNA was eluted in 30–60 μl DEPC water and the quantity of RNA measured by spectral analysis using the Nanodrop Spectrophotometer. RNA quality was determined by separation of the RNA via capillary electrophoresis using the Agilent 2000 Bioanalyzer. Microarray analysis was performed using Human GE 4x44k v2 Microarray Kit. The individual breast cancer cell lines were compared to a mixed reference pool on a single slide in which the mixed pool RNA was labelled with cyanine-3 and the individual cell lines with cyanine-5. The mixed reference pool consisted of equal amounts of cRNA from the SKBR3-Par and SKBR3-L breast cancer cell lines. Microarray slides were read using an Agilent Scanner and the Agilent Feature Extraction software version 7.5 was used to calculate gene expression values. Extracted data was imported into Rosetta Resolver 7.1 to create expression profiles for each individual breast cell line experiment. Cluster analysis was performed in Resolver and cell line profiles were exported to Excel (Microsoft) for additional analysis of the distribution of gene expression changes between SKBR3-Par and SKBR3-L cell line response data.

We performed gene set enrichment analysis using DAVID but found limited informative results from our analysis. Due to the alterations in the apoptotic potential of the SKBR3-L cells relative to their matched SKBR3-Par cells we used a list of 93 genes known to be involved in apoptosis (list obtained from the TaqMan® Human Apoptosis Array, Applied Biosystems). This list was used to screen the mRNA microarray data for apoptosis related genes which were up or down regulated by greater than 1.8-fold. All calculations were carried out using the limma library [13] of the open source R package (http://www.bioconductor.org).

### Terminal DNA transferase-mediated dUTP nick end labelling (TUNEL) assay

Cells were seeded in 24-well plates (3 × 10^4^/well) and incubated overnight at 37 °C, followed by addition of drug. After 72 h, media was collected and the wells washed once with PBS. Cells were trypsinised and added to the media collected for each sample. Cells were centrifuged at 300×g for 5 min and the media was aspirated. 150 μl of PBS was added, the pellet re-suspended and the total volume transferred to a round bottomed 96 well plate. 50 μL of 4% para-formaldehyde was added and mixed. Cells were incubated at 4 °C for 60 min. The plate was centrifuged at 300×g for 5 min and the supernatant aspirated leaving approximately 15 μL in each well. The remaining volume was used to resuspend the cells and 200 μL of ice cold 70% ethanol was added to each well. After fixing for 2 h at − 20 °C, the cells were stained according to the protocol for the Guava TUNEL assay (Millipore). Cells were analysed on the Guava EasyCyte (Millipore). Positive and negative controls were performed with each assay.

### Western blotting

Protein extraction was performed as previously described [12]. Protein (30 μg) in sample buffer (3 mM Tris HCl; 5% Sodium dodecyl sulphate (SDS); 12.5% beta-mercaptoethanol; 29% glycerol; 0.1% bromophenol blue) was heated to 95 °C for 5 min and proteins were separated on 7.5 or 10% gels (Lonza). The protein was transferred to Hybond-ECL nitrocellulose membrane (Amersham Biosciences). The membrane was blocked with 5% milk powder (Biorad) in 0.1% PBS-Tween at room temperature for 1 h, then incubated overnight at 4 °C in 1 μg/ml primary antibody for PARP (BD Biosciences), BAX (BD Biosciences), MCL-1 (BD-Biosciences), DR4, DR5, c-FLIP, BID (Cell Signalling), and α-tubulin (Sigma Aldrich) in 0.1% PBS-Tween with 5% milk powder. The membrane was washed three times with 0.5% PBS-Tween and then incubated at room temperature with 1 μg/ml anti-mouse secondary antibody (Sigma Aldrich) or 0.3 μg/ml anti-rabbit secondary antibody (Sigma Aldrich) in 0.5% PBS-Tween with 5% milk powder for 1 h. The membrane was washed three times with 0.5% PBS-Tween followed by one wash with PBS alone. Detection was performed using Luminol (Santa Cruz Biotechnology) or ECL Advance (GE Healthcare). Densitometry for Western blotting analysis was performed using TotalLab TL100 version 2006 software (Nonlinear Dynamics).

### qRT-PCR analysis

Biological triplicates of each cell line variant were cultured and analysed as follows. Total RNA was isolated using RNeasy mini kit (Qiagen Ltd., Crawley, UK). cDNA was prepared from 500 ng RNA using oligo dT primers (MWG) and MMLV reverse transcriptase (Sigma-Aldrich). A 1:5 dilution of the cDNA was included in subsequent qPCR, performed using TaqMan® gene expression assays for FOXO3a (Hs00818121_m1). Expression of FOXO3a was normalised to the endogenous control gene GAPDH (Hs99999905_m1), identified as comparatively expressed in all of the cell line variants. qPCR was performed using a Mastercycler ep realplex2S system (Eppendorf, Cambridge, UK). The comparative CT method was used for data analysis [14].

### Caspase activation assays

1 × 10^4^ cells were seeded per well and the cells were allowed to adhere overnight. 100 ng/mL TRAIL was added to the treatment group versus untreated control. Caspase 3/7, 8, and 9 activity were measured after 8 h incubations, using the Apotox-Glo™ Triplex assay, Caspase-Glo® 8, and Caspase-Glo® 9 assay kits and the GloMax®-Multi Microplate Multimode Reader (kits and equipment from Promega). Caspase 3/7, 8 and 9 activities were normalised against cell viability, which was determined using the GF-AFC Substrate Live Cell Assay (Triplex assay kit). Treatments were carried out using triplicate wells in each of three independent experiments.

### Statistical analysis

IC_50_ values were calculated using CalcuSyn software (BioSoft). The Student’s *t* test was used to compare levels of cleaved PARP and total PARP and to compare the expression of protein levels of BAX and MCL-1 between parental and resistance cell lines. The Students t-test was also used to compare the expression of mRNA and protein levels between parental and resistant cell lines and to compare differences in TRAIL induced apoptosis. *P* < 0.05 was considered statistically significant.

## Results

### Lapatinib does not induce apoptosis in cell line models of acquired lapatinib resistance

**Fig. 1:**
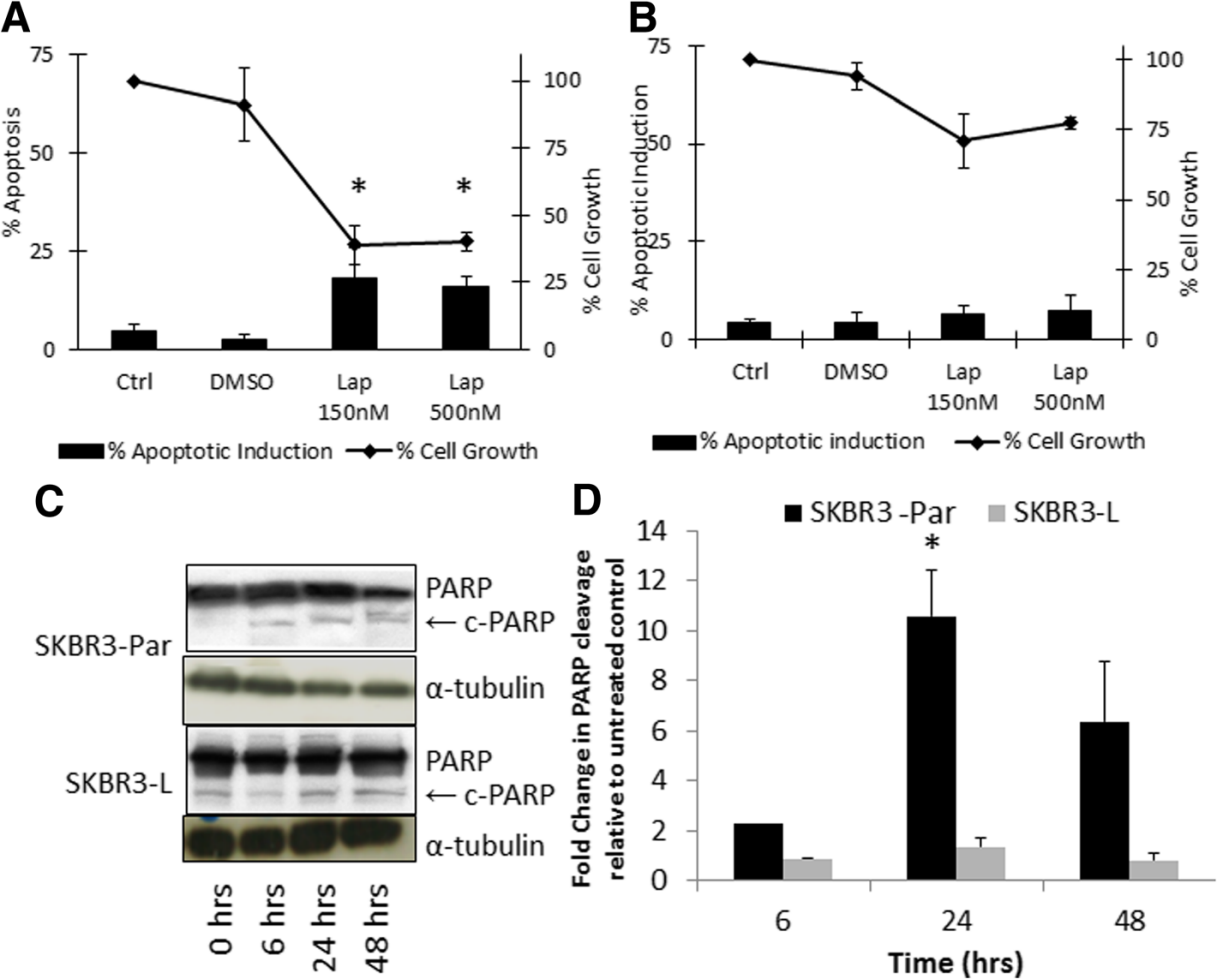
Percentage apoptosis induction measured by the TUNEL assay and percentage cell growth measured using Viacount on the Guava EasyCyte following treatment with either DMSO or lapatinib at 150 nM and 500 nM for 72 h in **a**) SKBR3-Par or **b**) SKBR3-L cells. **c**) Western blot for total and cleaved PARP following treatment with 150 nM lapatinib for 6, 24 and 48 h. **d**) fold change in cleaved PARP (relative to total PARP) as measured by densitometry relative to untreated control. Error bars represent the standard deviation of triplicate independent experiments. ‘*’ indicates a *p* value of < 0.05 calculated by Student’s t-test

We previously established two cell line models of acquired resistance to lapatinib, SKBR3-L (lapatinib IC_50_ value = 6.5 ± 0.4 μM) and HCC1954-L (lapatinib IC_50_ value = 2.7 ± 0.1 μM) [12]. In SKBR3-Par cells, lapatinib (500 nM) induces significant apoptosis (15.8 ± 2.0%) compared to untreated control cells (4.6 ± 2.7%) (*p* < 0.05), and inhibits cell growth by 60.8 ± 7.3% (Fig. 1a). In SKBR3-L cells, lapatinib (500 nM) did not induce significant apoptosis compared to the untreated controls (Fig. 1b), and only reduced proliferation by 22.6 ± 1.9%. Lapatinib treatment also caused a significant increase in the level of cleaved PARP in SKBR3-Par cells (*p* = 0.002) but not in SKBR3-L cells (Fig. 1c, d).

### MCL-1 and BAX expression are altered in lapatinib resistant cells

**Fig. 2:**
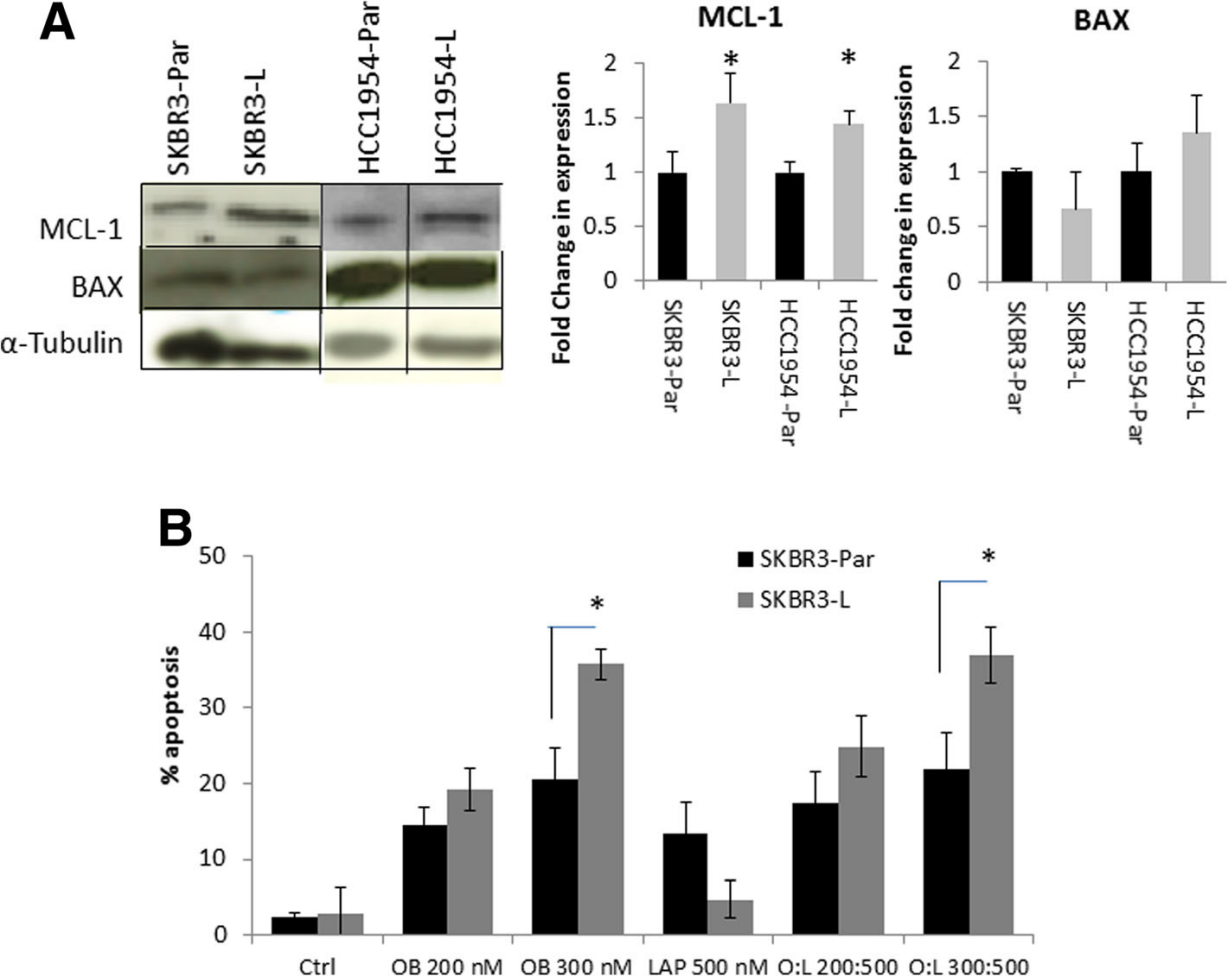
**a** A composite of representative images of MCL-1 and BAX expression measured by Western blotting. Densitometry analysis relative to alpha-tubulin in SKBR3-Par, SKBR3-L, HCC1954-Par and HCC1954-L cell lines. ‘*’ indicates a significant increase (*p* < 0.05) in protein expression relative to the matched parental controls. **b** Percentage apoptosis induced by obatoclax and/or lapatinib (72 h) in SKBR3-Par and SKBR3-L cells, measured by the TUNEL assay. Error bars represent the standard deviation of triplicate independent experiments. ‘*’ indicates a p value of < 0.05 as calculated by Student’s t-test when comparing obatoclax 300 nM between SKBR3-Par and SKBR3-L cells or when comparing obatoclax (300 nM) and lapatinib (500 nM) between SKBR3-Par and SKBR3-L cells

In order to investigate potential alterations in apoptosis pathways that may contribute to resistance to lapatinb-induced apoptosis, we examined changes in expression of apoptosis related genes in SKBR3-L cells compared to SKBR3-Par cells. Based on microarray gene expression data (Additional file 7: Table S1) the anti-apoptotic protein MCL-1 is up-regulated 1.82-fold, while pro-apoptotic BAX expression is down-regulated 3.17-fold in SKBR3-L cells (Additional file 7: Table S1). Using Western blotting, we confirmed that MCL-1 protein levels are significantly increased in the SKBR3-L compared to SKBR3-Par cells (1.6-fold, *p* = 0.035), and BAX protein levels were slightly reduced in SKBR3-L (1.5-fold, (*p* = 0.058)) but the result was not significant (Fig. 2a). In HCC1954-L cells, MCL-1 protein levels were increased by 1.4 fold (*p* = 0.039), whilst BAX protein levels were unchanged (Fig. 2a). To determine if changes in MCL-1 and BAX expression occur in human HER2-amplified breast cancers treated with lapatinib we examined MCL-1 and BAX mRNA expression in breast cancer biopsies obtained pre- and post-21 days of lapatinib treatment using RNAseq data obtained from the TCHL neoadjuvant clinical trial (ICORG 10–05; NCT01485926) [15] (Additional file 6: Methods). RNAseq data was available for 1 tumour treated with lapatinib in combination with chemotherapy and 3 tumours treated with lapatinib in combination with trastuzumab and chemotherapy. Of the 4 lapatinib-treated tumours, 2 showed an increase in MCL-1 and a decrease in BAX mRNA levels post-treatment (Table 1), similar to the changes observed in the SKBR3-L cells. Of 4 tumours treated with trastuzumab plus chemotherapy, an increase in MCL-1 and decrease in BAX was observed in 1 case. Therefore, we have demonstrated that changes which we observed in-vitro are also present in a subset of human breast cancers which were clinically treated with lapatinib or trastuzumab based regimens.

**Table 1:** Relative MCL-1 and BAX mRNA expression levels post-treatment (21 days) with docetaxel (T), carboplatin (C), trastuzumab (H) and/or lapatinib (L) in HER2-positive breast tumours^15^

Pt no.	Treatment regimen	Fold change in mRNA post-treatment	
		MCL-1	BAX
TCHL_39	TCH	1.1	0.9
**TCHL_45**	**TCH**	**1.6**	**0.7**
TCHL_50	TCH	1.0	0.8
TCHL_6	TCH	1.4	1.2
TCHL_29	TCL	1.0	1.2
TCHL_44	TCHL	0.9	1.1
**TCHL_54**	**TCHL**	**1.6**	**0.7**
**TCHL_7**	**TCHL**	**1.3**	**0.8**

Tumours with an increase in MCL-1 (≥1.2) and a decrease in BAX (≤0.8) are highlighted in bold

### Obatoclax induces apoptosis in lapatinib resistant cells

As the increased expression of MCL-1 in the SKBR3-L cells is likely to contribute to apoptosis resistance and MCL-1 is targeted by the small molecule inhibitor, obatoclax, we examined sensitivity to obatoclax in the lapatinib resistant cells. Using the TUNEL assay, we observed that obatoclax (300 nM) induced significantly greater apoptosis in SKBR3-L cells (35.7 ± 2.0%) compared to SKBR3-Par cells (20.5 ± 4.3%) (*p* = 0.013) (Fig. 2b) and in HCC1954-L cells (69.1 ± 1.9%) compared to HCC1954-Par cells (47.5 ± 1.1) (*p* = 0.005) (Additional file 1: Figure S1). Combining obatoclax with lapatinib did not significantly enhance apoptosis induction compared to obatoclax alone in either the SKBR3-Par or HCC1954-Par cell lines (Fig. 2b, Additional file 1: Figure S1).

### SKBR3-L cells show enhanced sensitivity to TRAIL

**Fig. 3:**
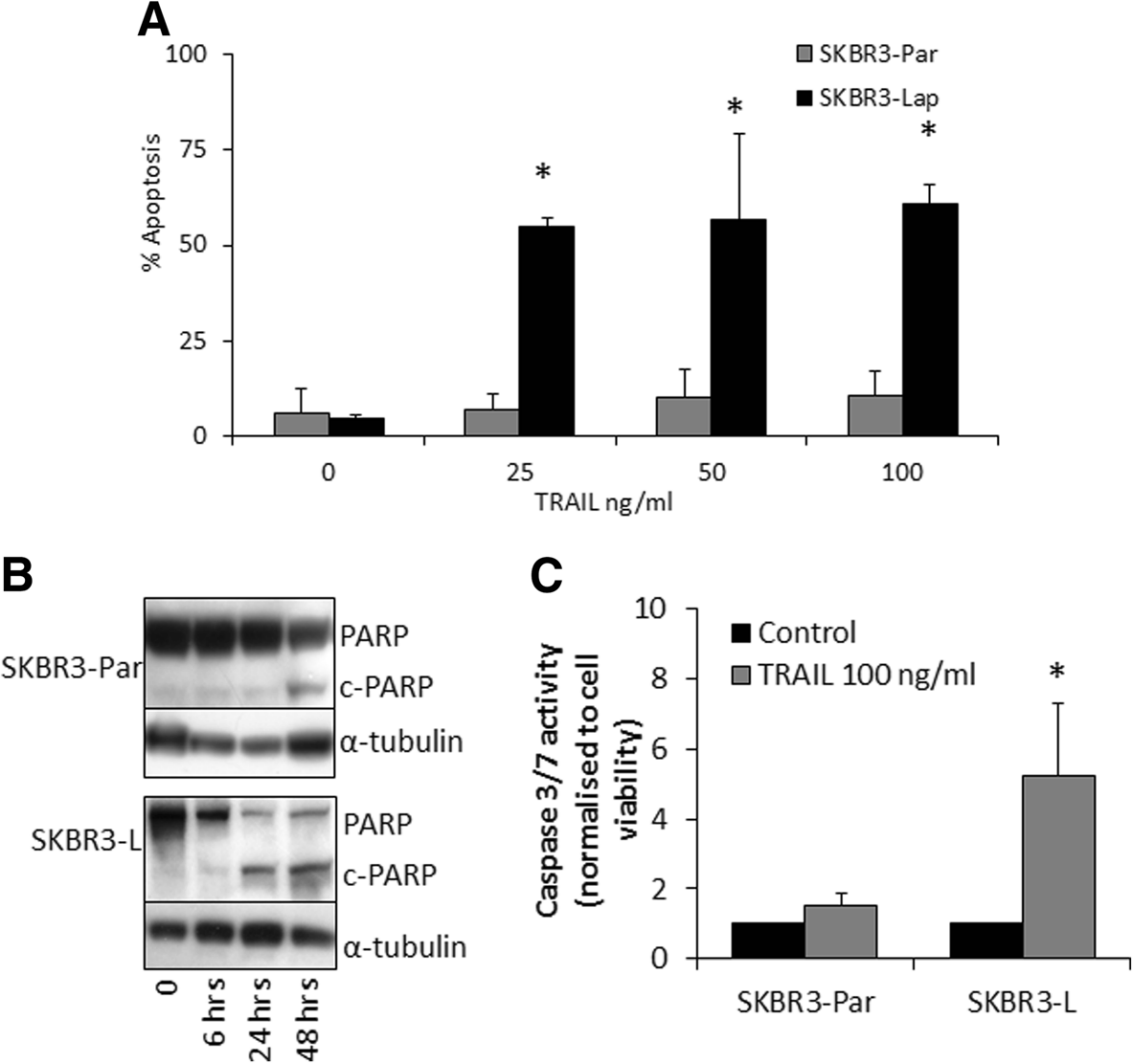
**a** Percentage apoptosis induction following 72 h treatment with increasing concentrations of TRAIL in SKBR3-Par and SKBR3-L cells. ‘*’ indicates a significant increase (*p* < 0.05 as calculated by Student’s t-test) in TRAIL induced apoptosis between SKBR3-Par and SKBR3-L cells at the relevant concentration. **b** Western blot analysis of PARP cleavage relative to total PARP following treatment with 25 ng/mL TRAIL for 6, 24 and 48 h in SKBR3-Par and –L cells. **c** The effect of 8-h of TRAIL (100 ng/ml) treatment on caspase 3/7 activity normalised to cell viability relative to untreated controls in SKBR3-Par and SKBR3-L cells. Error bars represent the standard deviation of triplicate independent experiments. ‘*’ indicates a significant increase (*p* < 0.05 as calculated by Student’s t-test) in Caspe3/7 induction by TRAIL in SKBR3-L cells relative to SKBR3-Par cells

In the gene expression data, increased expression of TRAIL-1 receptor (1.75-fold) and decreased expression of c-FLIP (1.6-fold) were noted in the SKBR3-L cells (Additional file 7: Table S1). Overexpression of FLICE-inhibitory protein (FLIP), an endogenous caspase-8 inhibitor, has been shown to protect cells against TRAIL-induced apoptosis. FLIP in turn can be regulated via the PI3-kinase/Akt pathway; Akt down regulation results in decreased levels of FLIP. Therefore, we examined sensitivity to TRAIL-induced apoptosis in the lapatinib resistant cells. TRAIL ligand (25 ng/ml) induced significantly greater apoptosis in SKBR3-L cells (54.7 ± 2.6%) than SKBR3-Par (7.0 ± 4.0%) (*p* < 0.01) cells, using the TUNEL assay (Fig. 3a). These results were confirmed by increased PARP cleavage (Fig. 3b; Additional file 2: Figure S2A) and activation of caspase 3/7 (Fig. 3c) following TRAIL treatment. Proliferation assays confirmed that TRAIL treatment significantly inhibited growth of SKBR3-L cells with an IC_50_ value of 2.6 ± 1.7 ng/ml (Additional file 2: Figure S2B). In SKBR3-Par cells, the highest concentration of TRAIL tested (100 ng/ml) achieved only 21.0 ± 3.2% growth inhibition (Additional file 2: Figure S2B). The SKBR3-L cells also showed enhanced sensitivity to TNF-α compared to the SKBR3-Par cells (Additional file 2: Figure S2C). However, neither the HCC1954-Par nor the HCC1954-L cells showed sensitivity to TRAIL treatment (Additional file 2: Figure S2D).

**Fig. 4:**
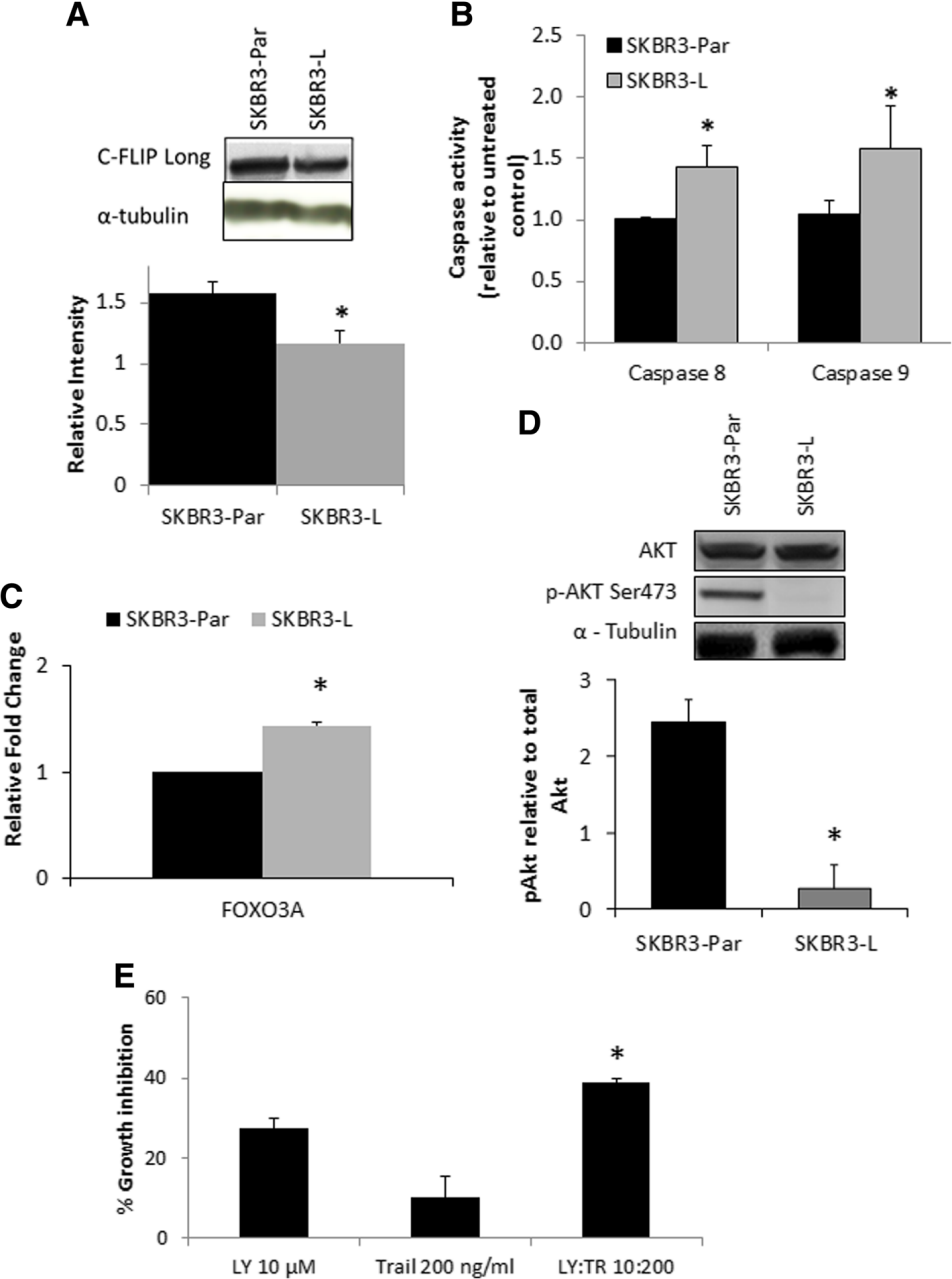
**a** Western blot and densitometry for c-FLIP long in SKBR3-Par and SKBR3-L cells. C-FLIP short was not detectable by Western blotting in either SKBR3-Par or SKBR3-L cells. **b** The effect of 8-h of TRAIL treatment (100 ng/ml) on caspase 8 and caspase 9 activity normalised to cell viability, relative to untreated controls in SKBR3-Par and SKBR3-L cells. **c** FOXO3a qRT-PCR in SKBR3-Par and SKBR3-L cells. **d** Western blot and densitometry for p-AKT (s473) relative to total AKT in SKBR3-Par and lapatinib resistant cell lines. **e** Percentage growth inhibition by LY294002 (LY) and/or TRAIL (TR) (72 h) in SKBR3-Par cells. Error bars represent the standard deviation of triplicate independent experiments. ‘*’ indicates a *p* value of < 0.05 as calculated by Student’s t-test

Despite the increase in TRAIL-1 receptor mRNA observed in the microarray gene expression data (Additional file 7: Table S1) neither TRAIL-1 and TRAIL-2 receptor expression were significantly altered in the SKBR3-L cells relative to SKBR3-Par cells based on flow cytometry and immunoblotting (Additional file 3: Figure S3). However, c-FLIP (long) showed a small but significant decrease, of 1.35 fold, in SKBR3-L cells relative to SKBR3-Par cells (*p* = 0.006) (Fig. 4a). TRAIL-induced apoptosis was associated with a significant increase in caspase 8 activity (1.4-fold, *p* = 0.014) and caspase 9 activity (1.6-fold, *p* = 0.04) in SKBR3-L cells (Fig. 4b).

### TRAIL sensitivity is associated with loss of p-AKT in SKBR3-L cells

The transcription factor FOXO3a has been implicated in regulating expression of c-FLIP and TRAIL-induced apoptosis [16]. In addition, lapatinib treatment has been implicated in increasing FOXO3a expression levels, via inhibition of p-AKT [17]. In SKBR3-L cells, we detected a significant increase in FOXO3a mRNA expression (1.4-fold, *p* < 0.01) (Fig. 4c) and an associated decrease in p-AKT levels (9.1-fold, *p* = 0.001) compared to SKBR3-Par cells (Fig. 4d). In contrast, HCC1954-L cells show a 2-fold increase in p-AKT levels compared to HCC1954-Par cells (p = 0.04) (Additional file 4: Figure S4A). Using the pan-PI3K kinase inhibitor LY294002 (10 μM) in combination with TRAIL, we demonstrated that inhibition of PI3K/AKT signalling for 1 h sensitised SKBR3-Par cells to subsequent treatment with TRAIL (100 ng/ml) (Fig. 4e).

We propose a mechanism of increased TRAIL sensitivity in the SKBR3-L cells, which results from a reduction of AKT phosphorylation, which subsequently leads to increased FOXO3a expression that in turn reduces c-FLIP expression. Decreased c-FLIP facilitates increased caspase-8 activation, resulting in the induction of the mitochondrial pathway of apoptosis and increased caspase-9 activation (Additional file 5: Figure S5). To determine if alterations in p-AKT occur in vivo, following lapatinib treatment, we examined changes in p-AKT levels post-treatment (14-days) in HER2-amplified breast cancers from patients treated with pre-operative lapatinib versus trastuzumab versus both, without chemotherapy for 14 days in the LPT1090906 neoadjuvant clinical trial (NCT00524303) [18]. Based on RPPA data, of the 13 cases where pre- and post-treatment p-AKT levels were measured on fresh frozen biopsies, 8 (61.5%) showed increased p-AKT (> 1.2-fold), 3 (23.1%) showed no significant change and 2 (15.4%) showed a decrease in p-AKT levels following 2 weeks of treatment with lapatinib only (Table 2). Following two weeks of pre-operative lapatinib vs trastuzumab vs the combination of lapatinib and trastuzumab alone, patients were treated pre-operative with the same anti-HER2 agent(s) in combination with standard chemotherapy for 6 months and then underwent definitive breast and axilla surgery [18]. One of the two patients whose cancers had reduced p-AKT had a pathological complete response in breast and axillary lymph nodes. Interestingly 9/17 (59%) cancers that were treated with trastuzumab alone showed a significant decrease in p-AKT levels and 4/9 had a pCR, whereas only 2/9 (22.2%) tumours treated with trastuzumab and lapatinib showed decreased p-AKT post-treatment and both had a pCR.

**Table 2:** Change in phosphorylated AKT (pS473_AKT) post-treatment with lapatinib and/or trastuzumab in HER2-positive breast tumours^18^

Patient no.	Treatment regimen	pS473_AKT	
		Fold change post-treatment	pCR
21	Lapatinib	1.1	No
42	Lapatinib	1.3	No
3	Lapatinib	1.0	No
45	Lapatinib	1.5	No
85	Lapatinib	2.2	Yes
185	Lapatinib	1.6	No
369	Lapatinib	1.8	Yes
7	Lapatinib	1.7	No
8	Lapatinib	3.7	No
204	Lapatinib	1.3	No
**491**	**Lapatinib**	**0.7**	**No**
504	Lapatinib	1.1	Yes
**507**	**Lapatinib**	**0.7**	**Yes**
**81**	**Trastuzumab**	**0.5**	**Yes**
82	Trastuzumab	1.0	Yes
**321**	**Trastuzumab**	**0.4**	**Yes**
365	Trastuzumab	5.0	Yes
24	Trastuzumab	4.1	Yes
364	Trastuzumab	0.9	No
**5**	**Trastuzumab**	**0.6**	**Yes**
31	Trastuzumab	0.9	Yes
**84**	**Trastuzumab**	**0.6**	**No**
**202**	**Trastuzumab**	**0.5**	**No**
**367**	**Trastuzumab**	**0.4**	**Yes**
**6**	**Trastuzumab**	**0.6**	**No**
**39**	**Trastuzumab**	**0.3**	**No**
193	Trastuzumab	3.9	No
412	Trastuzumab	3.7	Yes
**442**	**Trastuzumab**	**0.2**	**No**
506	Trastuzumab	1.2	Yes
23	Trast + Lapatinib	4.1	Yes
44	Trast + Lapatinib	9.0	No
30	Trast + Lapatinib	2.7	Yes
**363**	**Trast + Lapatinib**	**0.5**	**Yes**
**303**	**Trast + Lapatinib**	**0.7**	**Yes**
88	Trast + Lapatinib	1.2	Yes
221	Trast + Lapatinib	1.1	Yes
373	Trast + Lapatinib	0.9	Yes
501	Trast + Lapatinib	1.3	Yes

Tumours with a decrease in pS473_AKT (≤0.8) are highlighted in bold

### Combining TRAIL and obatoclax increase growth inhibition in SKBR3-L cells

**Fig. 5:**
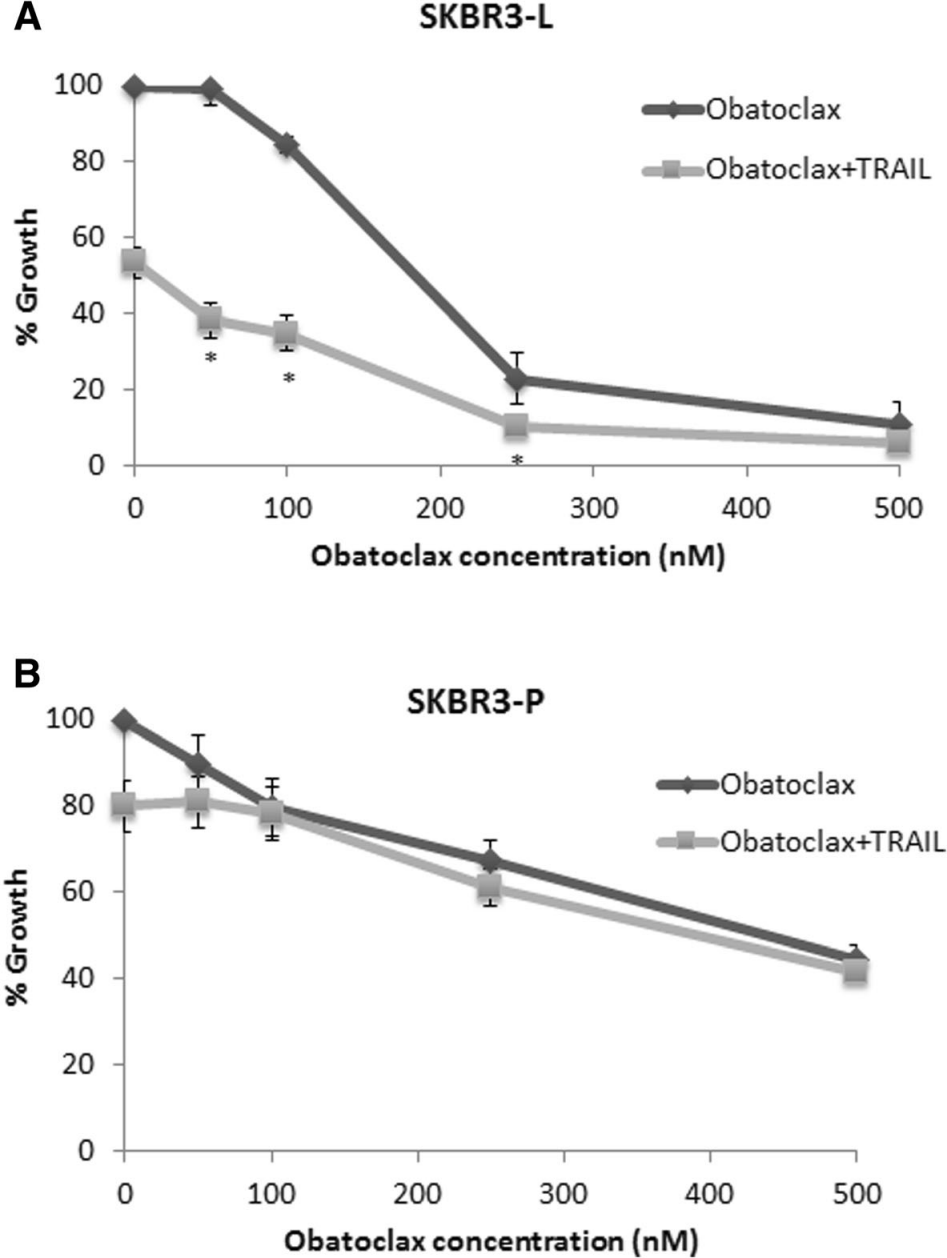
The effect of TRAIL ligand (5 ng/mL) in combination with obatoclax on proliferation of (**a**) SKBR3-L and (**b**) SKBR3-Par cells in a 5-day proliferation assay. Error bars represent the standard deviation of triplicate independent experiments. ‘*’ indicates a p value of < 0.05 as calculated by Student’s t-test

Previous studies have shown that obatoclax sensitises cancer cells to TRAIL-induced apoptosis [19, 20]. Therefore, we tested combined treatment with obatoclax and TRAIL to determine if this combination regimen could improve response in lapatinib resistant cells. The combination of TRAIL ligand (5 ng/mL) and obatoclax (50–250 nM) enhanced growth inhibition compared to either TRAIL or obatoclax alone in SKBR3-L cells (Fig. 5a) but not in SKBR3-Par cells (Fig. 5b). In the TRAIL resistant HCC1954-L cells, combined treatment did not improve response compared to obatoclax alone (Additional file 4: Figure S4B). We did not perform in vivo testing because of the lack of an appropriate in vivo model available to test the combination of obatocax and TRAIL ligands. This is because the SKBR3 cells do not form tumours in mice and although the HCC1954-L cells are tumorigenic they are not sensitive to TRAIL alone or in combination with obatoclax.

## Discussion

Lapatinib has clinical efficacy in the treatment of trastuzumab-refractory HER2-positive breast cancer, however, a significant proportion of patients develop progressive disease due to either innate or acquired resistance to lapatinib [21–23]. A number of potential mechanisms of acquired lapatinib resistance have been proposed including increased oestrogen receptor (ER) signalling [17] and increased SRC kinase activity [24]. However we observed no significant increase in either ER signalling or SRC kinase phosphorylation in the SKBR3-L or HCC1954-L cells [12, 25].

We and others have previously shown that lapatinib induces apoptosis in the lapatinib sensitive cell lines SKBR3 and BT474 [2, 3], however in this study we demonstrate that lapatinib fails to induce apoptosis in the HCC1954 cells. O’Brien et al. demonstrated that lapatinib has greater anti-proliferative effects in the SKBR3 rather than the HCC1954 cells, and results in the decrease of p-AKT (S473) in SKBR3 but not HCC1954 cells, likely indicating why lapatinib is not as effective in HCC1954 cells [26]. In this study, we showed that lapatinib failed to induce significant apoptosis in cells with acquired lapatinib resistance. Therefore, we examined alterations in apoptosis signalling in our lapatinib resistant models. Using gene expression profiling of apoptosis-related genes we observed that acquired lapatinib resistance was associated with changes in expression of BCL-2 family members.

BCL-2 family members play both pro- and anti-apoptotic roles in cell death and have been associated with acquired resistance to lapatinib in colon cancer [6]. Thus targeting this family of proteins may have therapeutic benefit in lapatinib resistant HER2-positive breast cancer. The BH3 mimetic obatoclax (GX15–070), which inhibits MCL-1, has shown activity in pre-clinical models of both colon and breast cancer [6, 8] including SKBR3 and BT474 cell lines. In vitro and in vivo studies reveal that both short and long term growth assays with obatoclax enhanced the toxicity of lapatinib [9, 27, 28]. In vitro knockdown of MCL-1 and BCL-X_L_ also enhanced the lethality of lapatinib in breast cancer cells [9].

In the SKBR3-L cells, expression of anti-apoptotic MCL-1 was increased relative to BAX expression and hence pushes the cells into a more anti-apoptotic/pro-survival phenotype, which may contribute to resistance to lapatinib-induced apoptosis. Obatoclax induced higher levels of apoptosis in the SKBR3-L cells than in the SKBR3-Par cells. To determine if sensitivity to obatoclax is a common feature of acquired lapatinib resistance, we tested another model of lapatinib resistance. We found that HCC1954-L cells also have increased expression of MCL-1, but no significant alteration in BAX expression. HCC1954-L cells did show enhanced sensitivity to obatoclax (apoptotic induction) compared to the HCC1954-Par cells. However, HCC1954-L cells were not more sensitive to the combination of obatoclax and lapatinib. Therefore, increases in MCL-1 expression are likely associated with sensitivity to obatoclax, but not to the combination of drugs.Using RNAseq data from the neoadjuvant TCHL trial [15], we have shown that 2 of the 4 tumours treated with lapatinib and/or trastuzumab and chemotherapy showed an increase in MCL-1 and a decrease in BAX mRNA levels following 21 days of treatment suggesting that the changes in the MCL-1/BAX ratio that we observed in our cell line model of acquired lapatinib resistance can occur in HER2-positive tumours. Thus, alterations in BCL-2 family members are implicated in lapatinib and trastuzumab resistance both in vitro and in vivo, providing a strong rationale for targeting the BCL-2 family in HER2-positive breast cancer. In regard to selecting patients for obatoclax treatment, MCL-1 expression could be determined by immunohistochemistry when comparing a patient's MCL-1 expression in their surgical biopsy compared to their most recent relapse biopsy sample, where an increase in MCL-1 expression is a criterion for inclusion.

Using an in vivo model of HER2-positive breast cancer Zoeller et al. [29] recently showed that matrix-attached cells are resistant to lapatinib, whereas the remaining tumour cells are sensitive. The resistant matrix-attached cells have increased levels of pro-apoptotic BCL-2. Cruickshanks [27] also demonstrated that anoikis-resistant breast cancer cells had multiple defects in their ability to undergo cell death processes, partly associated with increased expression of c-FLIP or protective BCL-2 proteins. In addition, anoikis-resistant cells had increased activity within the PI3K/AKT pathway. Interestingly though the authors found that inhibition of the PI3K/AKT pathway partially restored the sensitivity of anoikis resistant cells to treatment with lapatinib and obatoclax.

To date obatoclax has been tested in Phase I and Phase II clinical trials in leukaemia and lung cancers [29, 30]. A randomized phase II study of carboplatin and etoposide with or without obatoclax in small cell lung cancer demonstrated that the addition of obatoclax showed a trend towards better overall response rates [31]. ABT-263 (Navitoclax) the orally active analogue of ABT-737 is a BCL-2 targeting agent currently in Phase II studies in small cell lung cancer and chronic lymphocytic leukaemia. ABT-737 targets Bcl-2 and Bcl-X_L_, but not MCL-1, and MCL-1 can mediate resistance to ABT-737 [32]. Two further novel MCL-1 inhibitors MIK665 and S64315 are undergoing Phase I clinical trials in patients with haematological malignancies (NCT02992483, NCT02979366) but to date no preclinical studies have been published. Our studies provide a rationale for testing MCL-1 inhibitors such as obatoclax in patients with HER2-positive breast cancer following progression on lapatinib treatment.

Increased expression of MCL-1 has been implicated in resistance to TRAIL induced apoptosis in several cancer types [33–35]. However, in addition to enhanced sensitivity to obatoclax, we also observed significantly increased sensitivity to TRAIL-induced apoptosis in the lapatinib-resistant SKBR3-L cells. Dolloff et al., (2011) showed that pre-treatment of a panel of TRAIL-resistant colon cancer cell lines with lapatinib sensitized the cells to TRAIL, resulting in caspase activation and cell death [11]. Dolloff et al, attributed the increased TRAIL sensitivity to increased TRAIL receptor expression, which was mediated by activation of the transcription factor c-JUN [11]. Despite an observed increase in sensitivity to TRAIL in SKBR3-L cells, we found that TRAIL-1, / -2 receptor expression was low in the SKBR3-Par cells, and was not significantly increased in SKBR3-L cells indicating that TRAIL receptor expression was not associated with sensitivity to TRAIL treatment.

c-FLIP is a negative regulator of caspase-8 activation at the DISC complex of the TRAIL receptor [36]. Decreases in c-FLIP expression have been associated with increased caspase-8 activation and increased cell death. Decreased c-FLIP has been reported in ovarian and glioma cells treated with TRAIL [36, 37] or when the PI3K/AKT pathway is inhibited [37]. In the SKBR3-L cells the result of continuous exposure to lapatinib was an observed decrease in c-FLIP_L_ expression and a decrease in p-AKT levels relative to the SKBR3-Par cells. The reduction of p-AKT levels was not observed in HCC1954-Par cell line models. Dubska et al [38] showed that trastuzumab treatment of BT474 cells resulted in the cells having decreased death receptor expression and decreased TRAIL mediated cell death [38]. However, in SKBR3 cells, treatment with trastuzumab resulted in increased sensitivity to TRAIL, which they associated with inhibition of p-AKT [38]. To date several studies have found that inhibition of the PI3K pathway can result in acquired sensitivity to TRAIL in multiple cancer types [39–43]. Our results show that acquired lapatinib resistance in SKBR3-L cells results in a significant reduction in p-AKT(s473), which does not occur in HCC1954-Par cells likely indicating that loss of p-AKT(s473) is a factor in acquired TRAIL sensitivity.

AKT is a negative regulator of two transcription factors c-JUN [44] and FOXO3a [45]. c-JUN expression which is negatively regulated by p-AKT, has been shown to control the expression of the TRAIL-1 and TRAIL-2 death receptors [11]. Inhibition of p-AKT has previously been shown to increase TRAIL receptor expression in colon cancer and acute myelogenous leukaemia cells [11, 43, 46], which was associated with increased c-JUN expression [11, 46]. We observed that c-JUN mRNA expression was decreased in SKBR3-L relative to SKBR3-Par cells (data not shown). These results combined with our observation that TRAIL receptor levels are not increased in the SKBR3-L cells suggest that c-JUN mediated over-expression of TRAIL receptors does not play a role in the enhanced sensitivity to TRAIL observed in the SKBR3-L cells.

FOXO3a expression is significantly increased in the SKBR3-L cells relative to SKBR3-Par cells. Clinically, administration of lapatinib has been shown to increase FOXO3a expression in breast tumours, and in vitro, lapatinib treatment increased FOXO3a expression in BT474 cells [17]. Increased FOXO3a expression has been previously shown to inhibit expression of c-FLIP [16, 47]. Reduced c-FLIP facilitates increased activation of caspase 8. Caspase-8 activation can also lead to activation of the intrinsic pathway of apoptosis via BID cleavage, which in turn results in increased in caspase-9 activity, activation of caspase-3/7 and ultimately cell death [48]. In TRAIL-treated SKBR3-L cells, we observed caspase-8 and 9 activation. Thus the proposed mechanism of increased TRAIL sensitivity in the SKBR3-L cells, is that decreased AKT signalling (reduced phosphorylation of AKT), results in increased expression of FOXO3a which reduces c-FLIP expression. Decreased c-FLIP facilitates increased caspase-8 activation, which may cause BID cleavage, resulting in the induction of the mitochondrial pathway of apoptosis and increased caspase-9 activation. Based on the microarray data, the SKBR3-L cells have increased expression of BID mRNA (Additional file 7: Table S1) which may also contribute to enhanced TRAIL mediated apoptosis in SKBR3-L cells. Consistent with our observations, Zang et al. reported that downregulating c-FLIP_L_ in SKBR3 cells sensitised the cells to TRAIL [49] .

Using RPPA data obtained from the LPT1090906 neoadjuvant clinical trial [18], only 2 of the 13 (15.4%) cancers treated with lapatinib alone had decreased p-AKT levels following 14 days of treatment. A higher proportion of cancers treated with trastuzumab alone (9/17, 59%) showed a decrease in p-AKT levels, whereas 2/9 (22.2%) tumours treated with trastuzumab and lapatinib showed decreased p-AKT post-treatment. Preclinical studies using lapatinib and/or trastuzumab to target HER2 signalling have demonstrated inhibition of AKT signalling [3]; therefore a reduction in p-AKT levels may be a marker of successful HER2 inhibition facilitating subsequent tumour cell death with chemotherapy and HER2-inhibition. It should be noted, however, that in the NCT00524303 trial, there was no correlation between a reduction in p-AKT levels and the likelihood of a patient achieving a pathological complete response. However, those patients whose cancers have reduced p-AKT following a brief treatment course with trastuzumab and/or lapatinib, may derive clinical benefit from subsequent TRAIL-agonist therapy.

TRAIL treatment is an attractive target for cancer as it has demonstrated selectivity for cancer cells, whilst not affecting ‘normal cells’. TRAIL agonists have shown anti-tumour effects in vitro and in vivo and have been tested as monotherapies in haematological malignancies, colorectal cancer and non-small cell lung cancer [50], with approximately 15% of patients treated with TRAIL agonists, having a long term response to TRAIL therapy. However, TRAIL agonists have not significantly improved overall survival or response rates [50], and to date clinical trials testing the combination of TRAIL agonists with chemotherapy have failed to improve either response rates or increased overall survival. This has led to many TRAIL targeted therapies being dropped from clinical development. However, the dramatic sensitisation to TRAIL that we observed in the lapatinib resistant SKBR3-L cells suggests that there may be a subset of treatment-refractory HER2-positive metastatic breast cancers which are sensitive to TRAIL targeted therapies. We have previously reported that approximately 10% of patients with metastatic HER2-positive breast cancer obtain a durable complete response following treatment with a trastuzumab-based regimen [51]. Although there are new treatment options, such as pertuzumab and T-DM1, which improve response, the majority of patients with metastatic HER2-positive breast cancer develop progressive disease and ultimately die. If TRAIL therapy offers the possibility of clinical benefit for a subset of these patients, it warrants further investigation. Interestingly, the new small molecule ONC201, which can induce the expression of TRAIL, has demonstrated good preclinical activity in some triple negative breast cancer cell lines which have elevated phosphorylation of retinoblastoma. ONC201 is currently in Phase II trials of neuroendocrine tumours, and represents a novel method to target TRAIL alone or in combination with other drugs [52]. The challenge is to identify clinical biomarkers that will enable selection of the patients who are most likely to benefit from TRAIL therapy. The molecular alterations that contribute to TRAIL sensitivity in the SKBR3-L cells, specifically decreased phosphorylation of AKT, increased FOXO3a expression and decreased cFLIP expression, may represent biomarkers for TRAIL sensitivity.

## Conclusions

In the SKBR3-L cells, combining the two apoptosis inducing treatments, obatoclax and TRAIL produced a greater response than the single agents. Thus, this combination regime could provide clinical benefit for lapatinib-refractory HER2-positive breast cancer. However, the combination did not produce an enhanced response in the HCC1954-L resistant model, consistent with the observation that HCC1954-Par cells do not show enhanced sensitivity to TRAIL and highlighting the importance of appropriate predictive biomarkers for obatoclax and/or TRAIL therapy, for example high MCL-1/BAX ratio and/or low p-AKT. Based on our data we believe that MCL-1 and TRAIL targeted therapies warrant further investigation in patients with treatment refractory HER2-positive breast cancer.

## Additional files

Additional file 1: **Figure S1.** Percentage apoptosis induction by obatoclax and/or lapatinib in HCC1954-Par and HCC1954-L cells. Percentage apoptosis induction by obatoclax (100, 200, 300 nM), and/or lapatinib (500 nM) in HCC1954-Par and HCC1954-L cells, measured by TUNEL assay. Error bars represent the standard deviation of triplicate experiments. ‘*’ indicates a *p* value of < 0.05 as calculated by Student’s t-test when comparing obatoclax alone between HCC1954-Par and HCC1954-L cells. (TIF 50 kb)

Additional file 2: **Figure S2.**The impact of TRAIL and TNF-alpha treatment in SKBR3-Par, -L and the impact of TRAIL in HCC1954-P and -L cells A) Densitometry analysis of PARP cleavage relative to total PARP following treatment with 25 ng/mL TRAIL for 6, 24 and 48 h in SKBR3-Par and –L cells. ‘*’ indicates a significant difference (*p* < 0.05 as calculated by students’ t-Test) when comparing TRAIL apoptosis induction between SKBR3-Par untreated and treated. Proliferation assays in SKBR3-Par and SKBR3-L treated with B) TRAIL or C) TNF alpha. D) Proliferation assays in HCC1954-Par and HCC1954-L cells treated with TRAIL. Error bars represent the standard deviation of triplicate independent experiments. (JPG 68 kb)

Additional file 3: **Figure S3.** TRAIL expression in SKBR3-Par and SKBR3-L cells. A) TRAIL 1 and TRAIL 2 receptor expression in SKBR3-Par, and SKBR3-L cells. B) Western blots for TRAIL 1 and TRAIL 2 receptor in SKBR3-Par and SKBR3-L cells. Median fluorescence intensity was used to compare receptor expression for parental and drug resistant lines. (TIF 63 kb)

Additional file 4: **Figure S4.** Targeting TRAIL in HCC1954-Par and -L cells. A) Western blot and densitometry for pAKT (Ser473) relative to total AKT in HCC1954-Par and HCC1954-L cells. Error bars represent the standard deviation of triplicate independent experiments. B) The effect of TRAIL ligand (25 ng/mL) in combination with obatoclax on proliferation of HCC1954-L. Error bars represent the standard deviation of triplicate independent experiments. ‘*’ indicates a p value of < 0.05 as calculated by Student’s t-test. (TIF 75 kb)

Additional file 5: **Figure S5.** Representative figure demonstrating hypothesised acquired sensitivity to TRAIL in SKBR3-L cells that have acquired resistance to lapatinib. Representative figure demonstrating hypothesised acquired sensitivity to TRAIL in SKBR3 cells that have acquired resistance to lapatinib. (TIF 125 kb)

Additional file 6: Supplementary materials and methods. Description and results of cell line fingerprinting, Flow cytometry workflow and details of the RNAseq analysis. (DOCX 16 kb)

Additional file 7: Expression data for differentially expressed apoptosis related genes in SKBR3 and SKBR3-L cells. Expression data for differentially expressed apoptosis related genes in SKBR3 and SKBR3-L cells (> 1.6-fold change in expression, *p* < 0.05). (DOCX 15 kb)
