# Supplementary figures and images for: Development of acquired resistance to lapatinib may sensitise HER2-positive breast cancer cells to apoptosis induction by obatoclax and TRAIL
Source: BMC Cancer. 2018 Oct 11;18:965. doi: 10.1186/s12885-018-4852-1 (PMC6180577; doi:10.1186/s12885-018-4852-1)

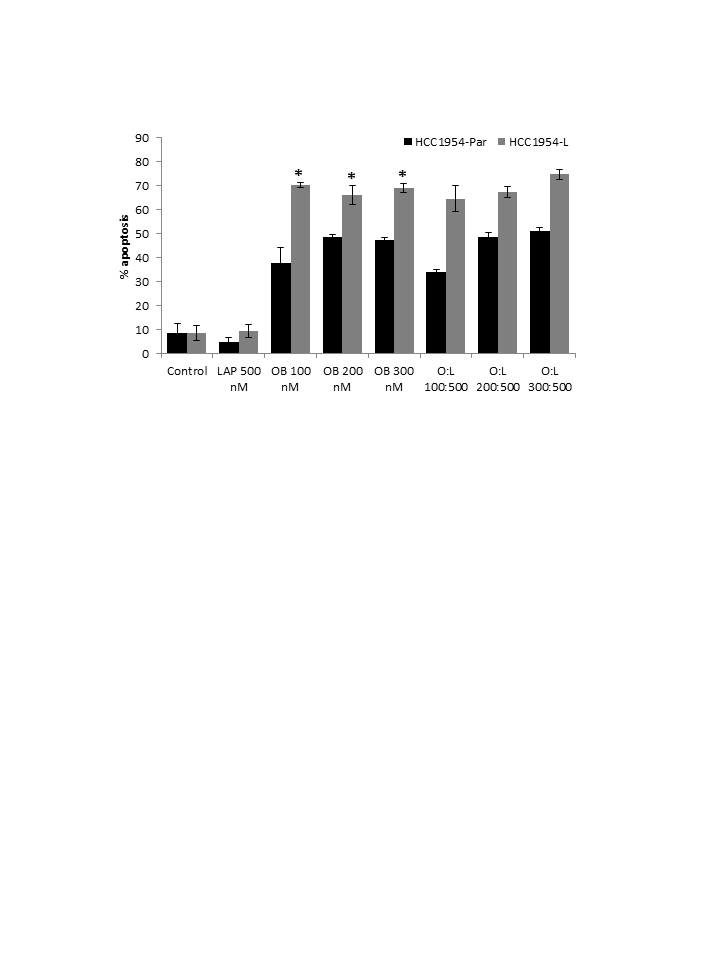

Supplement: Supplementary file 1 — Figure S1. Percentage apoptosis induction by obatoclax and/or lapatinib in HCC1954-Par and HCC1954-L cells. Percentage apoptosis induction by obatoclax (100, 200, 300 nM), and/or lapatinib (500 nM) in HCC1954-Par and HCC1954-L cells, measured by TUNEL assay. Error bars represent the standard deviation of triplicate experiments. ‘*’ indicates a p value of < 0.05 as calculated by Student’s t-test when comparing obatoclax alone between HCC1954-Par and HCC1954-L cells. (TIF 50 kb) [file 12885_2018_4852_MOESM1_ESM.tif]

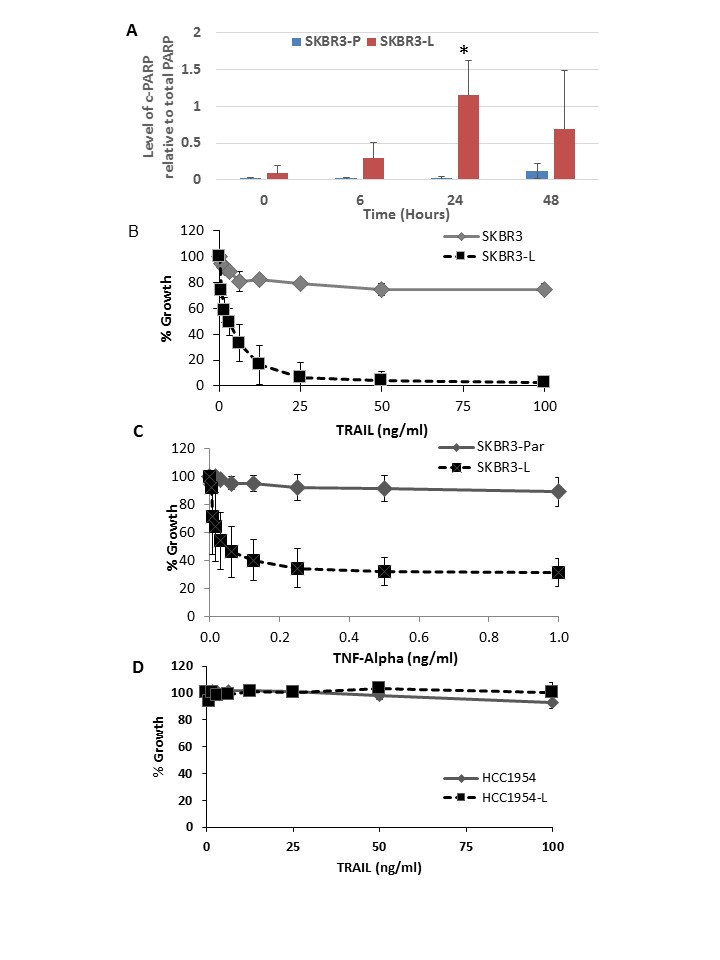

Supplement: Supplementary file 2 — Figure S2.The impact of TRAIL and TNF-alpha treatment in SKBR3-Par, -L and the impact of TRAIL in HCC1954-P and -L cells A) Densitometry analysis of PARP cleavage relative to total PARP following treatment with 25 ng/mL TRAIL for 6, 24 and 48 h in SKBR3-Par and –L cells. ‘*’ indicates a significant difference (p < 0.05 as calculated by students’ t-Test) when comparing TRAIL apoptosis induction between SKBR3-Par untreated and treated. Proliferation assays in SKBR3-Par and SKBR3-L treated with B) TRAIL or C) TNF alpha. D) Proliferation assays in HCC1954-Par and HCC1954-L cells treated with TRAIL. Error bars represent the standard deviation of triplicate independent experiments. (JPG 68 kb) [file 12885_2018_4852_MOESM2_ESM.jpg]

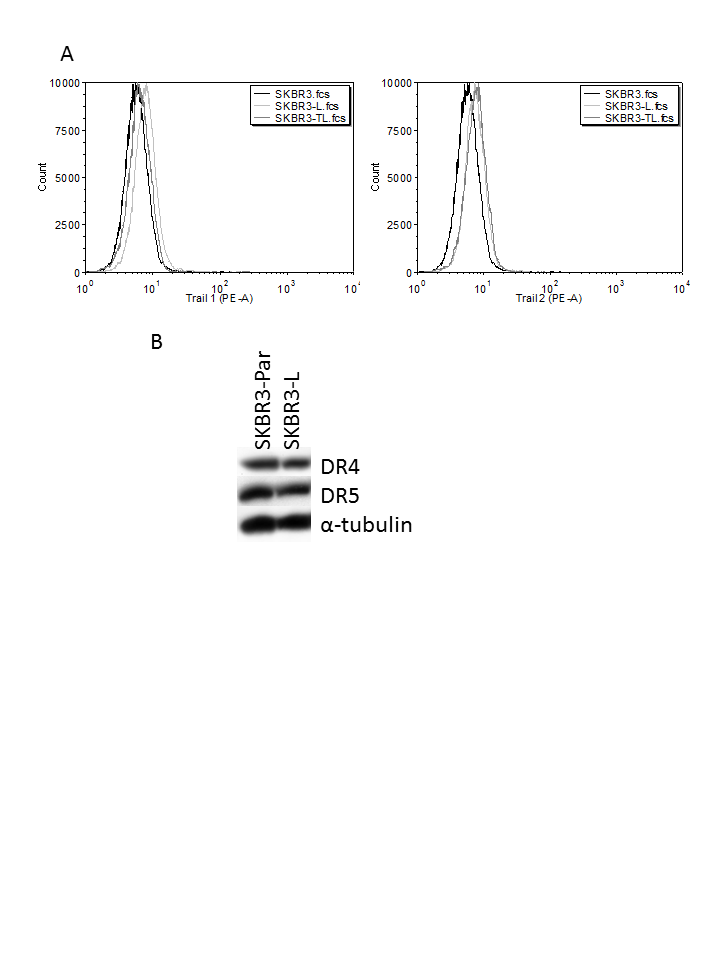

Supplement: Supplementary file 3 — Figure S3. TRAIL expression in SKBR3-Par and SKBR3-L cells. A) TRAIL 1 and TRAIL 2 receptor expression in SKBR3-Par, and SKBR3-L cells. B) Western blots for TRAIL 1 and TRAIL 2 receptor in SKBR3-Par and SKBR3-L cells. Median fluorescence intensity was used to compare receptor expression for parental and drug resistant lines. (TIF 63 kb) [file 12885_2018_4852_MOESM3_ESM.tif]

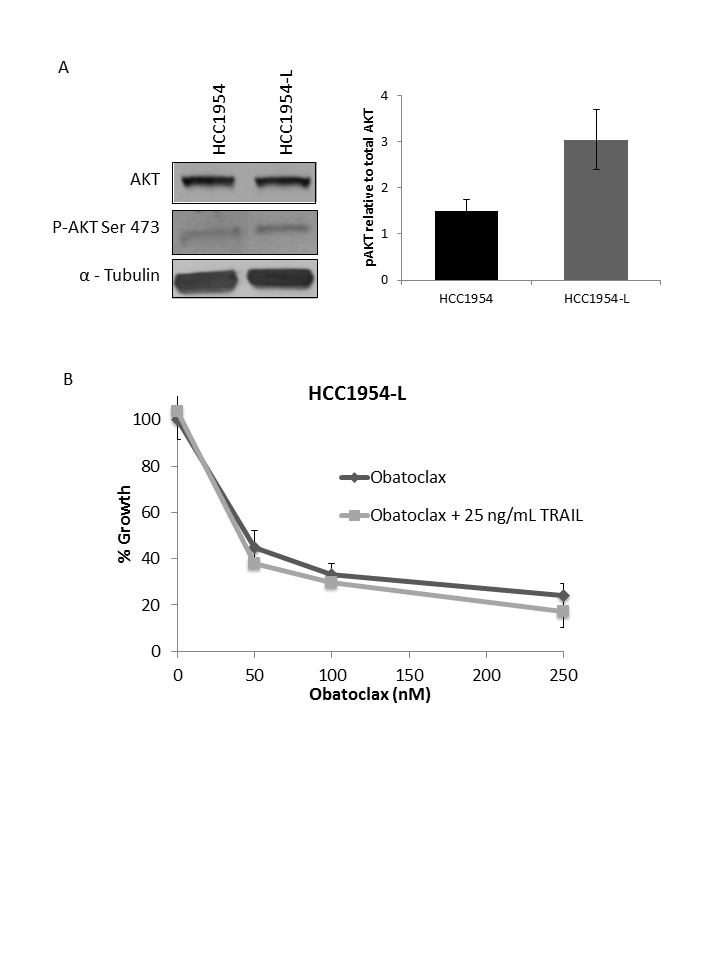

Supplement: Supplementary file 4 — Figure S4. Targeting TRAIL in HCC1954-Par and -L cells. A) Western blot and densitometry for pAKT (Ser473) relative to total AKT in HCC1954-Par and HCC1954-L cells. Error bars represent the standard deviation of triplicate independent experiments. B) The effect of TRAIL ligand (25 ng/mL) in combination with obatoclax on proliferation of HCC1954-L. Error bars represent the standard deviation of triplicate independent experiments. ‘*’ indicates a p value of < 0.05 as calculated by Student’s t-test. (TIF 75 kb) [file 12885_2018_4852_MOESM4_ESM.tif]

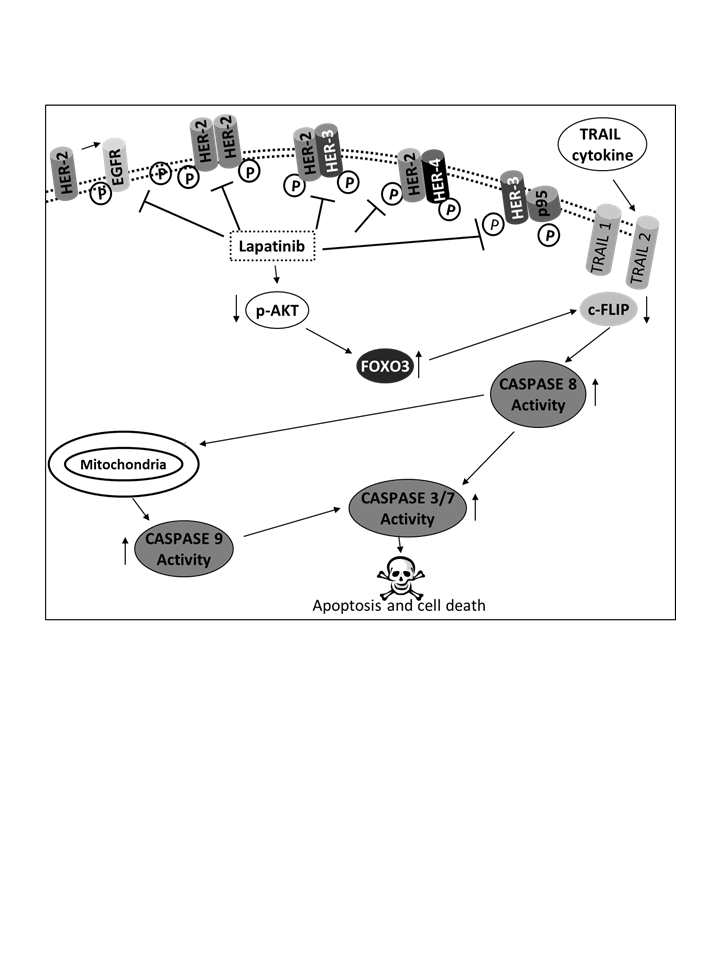

Supplement: Supplementary file 5 — Figure S5. Representative figure demonstrating hypothesised acquired sensitivity to TRAIL in SKBR3-L cells that have acquired resistance to lapatinib. Representative figure demonstrating hypothesised acquired sensitivity to TRAIL in SKBR3 cells that have acquired resistance to lapatinib. (TIF 125 kb) [file 12885_2018_4852_MOESM5_ESM.tif]
