# Supplementary material for: Development of acquired resistance to lapatinib may sensitise HER2-positive breast cancer cells to apoptosis induction by obatoclax and TRAIL
Source: BMC Cancer. 2018 Oct 11;18:965. doi: 10.1186/s12885-018-4852-1 (PMC6180577; doi:10.1186/s12885-018-4852-1)
Supplement: Supplementary file 6 — Supplementary materials and methods. Description and results of cell line fingerprinting, Flow cytometry workflow and details of the RNAseq analysis. (DOCX 16 kb) [file 12885_2018_4852_MOESM6_ESM.docx]

**Supplementary Methods**

**Cell culture**

Supplementary materials and methods 1: DNA fingerprint analysis of cell lines used in this study conducted by Source Bioscience.

| **Sample** | **Allele** | **AMEL** | **CSF1PO** | **D13S317** | **D16S539** | **D18S51** | **D21S11** | **D3S1358** | **D5S818** | **D7S820** | **D8S1179** | **FGA** | **Penta_D** | **Penta_E** | **TH01** | **TPOX** | **vWA** |
| --- | --- | --- | --- | --- | --- | --- | --- | --- | --- | --- | --- | --- | --- | --- | --- | --- | --- |
| **HCC1954-PAR** | **Allele 1** | X | 10 | 8 | 9 | 14 | 28 | 15 | 11 | 10 | 12 | 22 | 9 | 12 | 6 | 8 | 18 |
|  | **Allele 2** | X | 10 | 9 | 11 | 18 | 32.2 | 16 | 11 | 11 | 15 | 23 | 12 | 16 | 7 | 9 | 19 |
| **ATCC reference** | **Allele** | X | 10 | 8,9 | 9,11 | N/A | N/A | N/A | 11 | 10,11 | N/A | N/A | N/A | N/A | 6,7 | 8,9 | 18,19 |
| **HCC1954-LAP** | **Allele 1** | X | 10 | 8 | 9 | 18 | 28 | 15 | 11 | 10 | 12 | 22 | 9 | 12 | 7 | 8 | 18 |
|  | **Allele 2** | X | 10 | 9 | 11 | 18 | 32.2 | 16 | 11 | 11 | 15 | 23 | 12 | 16 | 7 | 9 | 19 |
| **ATCC reference** | **Allele** | X | 10 | 8,9 | 9,11 | N/A | N/A | N/A | 11 | 10,11 | N/A | N/A | N/A | N/A | 6,7 | 8,9 | 18,19 |
| **SKBR3-PAR** | **Allele 1** | X | 12 | 11 | 9 | 10 | 30 | 17 | 9 | 9 | 11 | 20 | 9 | 11 | 6 | 10 | 17 |
|  | **Allele 2** | X | 12 | 12 | 9 | 13 | 30.2 | 17 | 12 | 12 | 12 | 20 | 12 | 11 | 6 | 10 | 18 |
| **ATCC Reference** | **Allele** | X | 12 | 11,12 | 9 | N/A | N/A | N/A | 9,12 | 9,12 | N/A | N/A | N/A | N/A | 6 | 10 | 17,18 |
| **SKBR3-L** | **Allele 1** | X | 12 | 11 | 9 | 10 | 30 | 17 | 9 | 9 | 11 | 20 | 9 | 10 | 8 | 8 | 17 |
|  | **Allele 2** | X | 12 | 12 | 9 | 13 | 30.2 | 17 | 12 | 12 | 12 | 20 | 12 | 11 | 9 | 11 | 17 |
| **ATCC Reference** | **Allele** | X | 12 | 11,12 | 9 | N/A | N/A | N/A | 9,12 | 9,12 | N/A | N/A | N/A | N/A | 8,9 | 8,11 | 17,18 |

**Flow cytometry**

TRAIL 1 and 2 receptor expression was determined by staining cells with anti-human TRAIL R1 (TNFRSF10A/DR4)-Phycoerythrin and anti-human TRAIL R2 (TNFRSF10B/DR5)-Phycoerythrin (R&D Systems). Briefly, 1 x 10^6^ cells were trypsinised and resuspended in media for 6 hours to allow receptor expression to recover. Cells were then washed three times with phosphate-buffered saline (PBS) and fixed/permeabilised using 70% ice-cold ethanol and overnight incubation (-20°C). Cells were Fc blocked with 1:10 human IgG in PBS for 20 minutes and subsequently immunostained for one hour at room temperature. Following incubation, stained cells were washed twice and resuspended in 500 µl PBS. Samples were analysed using a FACS Aria flow cytometer (Becton Dickinson, San Jose, CA) and data analysis performed using FCS Express 4 software (De Novo Software, USA). Neutrophil cells were used as a positive control for TRAIL 1 and TRAIL 2 receptor expression.

**RNA-seq analysis**

RNA was extracted from 8 matched pairs of pre and on treatment samples from the TCHL trial. RNA was isolated using RNeasy mini kits (Qiagen) according to the manufacturer’s instructions. Quantity and purity were determined using a Bioanalyzer® (Agilent, Santa Clara, CA). Libraries were prepared using the Illumina TruSeq stranded mRNA library prep kit according to the manufacturer’s instructions (Illumina, San Diego, CA). Sequencing was performed using an Illumina HiSeq 2500, to produce 75 bp paired-end reads and quality control was conducted using FASTQC (<http://www.bioinformatics.babraham.ac.uk/projects/fastqc/>). The adapter sequences were trimmed using BBmap (<http://jgi.doe.gov/data-and-tools/bbtools/bb-tools-user-guide/bbmap-guide/>) and were aligned to human genome version 19, using the sequence aligner subread (1) via the bioconductor R package Rsubread (<https://bioconductor.org/packages/>). Next Samtools (2) and Picard tools (<http://broadinstitute.github.io/picard/>) were used to sort the alignments and remove duplicate reads respectively. The data was then scale normalised using the TMM (trimmed mean of M values (TMM)) normalisation method (3).

References:

1. Liao Y, Smyth GK, Shi W 2013 The Subread aligner: fast, accurate and scalable read mapping by seed-and-vote. Nucleic Acids Research 41 e108. doi: 10.1093/nar/gkt214.

2. Li H, Handsaker B, Wysoker A, Fennell T, Ruan J, Homer N, Marth G, Abecasis G, Durbin R, Genome Project Data Processing S. The Sequence Alignment/Map format and SAMtools. Bioinformatics. 2009; 25: 2078-9. doi: 0.1093/bioinformatics/btp352.

3. Robinson, M.D. and Oshlack, A 2010 A scaling normalization method for differential expression, Genome Biology 11 R25 doi:10.1186/gb-2010-11-3-r25
