# Supplementary material for: Development of acquired resistance to lapatinib may sensitise HER2-positive breast cancer cells to apoptosis induction by obatoclax and TRAIL
Source: BMC Cancer. 2018 Oct 11;18:965. doi: 10.1186/s12885-018-4852-1 (PMC6180577; doi:10.1186/s12885-018-4852-1)
Supplement: Supplementary file 7 — Expression data for differentially expressed apoptosis related genes in SKBR3 and SKBR3-L cells. Expression data for differentially expressed apoptosis related genes in SKBR3 and SKBR3-L cells (> 1.6-fold change in expression, p < 0.05). (DOCX 15 kb) [file 12885_2018_4852_MOESM7_ESM.docx]

**Supplementary table 1.** Expression data for differentially expressed apoptosis related genes in SKBR3 and SKBR3-L cells (>1.6-fold change in expression, p<0.05).

| **Primary Name** | **Description** | **Accession #** | **Fold Change** | **P-value** |
| --- | --- | --- | --- | --- |
| APAF1 | apoptotic peptidase activating factor 1 | NM_181861 | 1.68 | 0.00049 |
| BCL2L1 | BCL2-like 1 | NM_001191 | 1.64 | 0.00001 |
| **BID** | **BH3 interacting domain death agonist** | **NM_001196** | **1.66** | **0.00062** |
| **BAX** | **BCL2-associated X protein** | **NM_004324** | **-3.17** | **6.69E-11** |
| BBC3 | BCL2 binding component 3 | NM_014417 | -2.31 | 1.95E-07 |
| BBC3 | BCL2 binding component 3 | NM_014417 | -1.83 | 0.00007 |
| BCL2L1 | BCL2-like 1 | NM_001191 | 1.64 | 0.00001 |
| BNIP3L | BCL2/adenovirus E1B 19kDa interacting protein 3-like | NM_004331 | 1.75 | 0.00021 |
| BNIP3L | BCL2/adenovirus E1B 19kDa interacting protein 3-like | AL132665 | 1.90 | 0.00003 |
| CARD9 | caspase recruitment domain family, member 9 | NM_022352 | -3.70 | 1.84E-12 |
| CASP1 | caspase 1, apoptosis-related cysteine peptidase (interleukin 1, beta, convertase) | NM_033292 | -2.28 | 2.93E-07 |
| CASP4 | caspase 4, apoptosis-related cysteine peptidase | NM_033306 | -1.68 | 0.00053 |
| **CFLAR** | **CASP8 and FADD-like apoptosis regulator (c-FLIP)** | **BT006751** | **-1.64** | **0.0009** |
| CRADD | CASP2 and RIPK1 domain containing adaptor with death domain | NM_003805 | 2.38 | 8.89E-08 |
| HRK | harakiri, BCL2 interacting protein (contains only BH3 domain) | NM_003806 | -2.35 | 1.29E-07 |
| **MCL1** | **myeloid cell leukemia sequence 1 (BCL2-related)** | **NM_021960** | **1.82** | **0.00008** |
| NLRP12 | NLR family, pyrin domain containing 12 | NM_033297 | 1.74 | 0.00024 |
| NFKBIB | nuclear factor of kappa light polypeptide gene enhancer in B-cells inhibitor, beta | BC007197 | 1.62 | 0.0011 |
| RELB | v-rel reticuloendotheliosis viral oncogene homolog B | NM_006509 | -1.65 | 0.00073 |
| RIPK2 | receptor-interacting serine-threonine kinase 2 | NM_003821 | -1.68 | 0.00051 |
| TBK1 | TANK-binding kinase 1 | NM_013254 | 1.62 | 0.00111 |
| **TNFRSF10A** | **tumor necrosis factor receptor superfamily, member 10a (TRAILR-1)** | **NM_003844** | **1.75** | **0.00019** |
| TNFRSF1A | Homo sapiens tumor necrosis factor receptor superfamily, member 1A (TNFRSF1A), mRNA. | NM_001065 | 1.66 | 6.87E-06 |
| TNFRSF21 | tumor necrosis factor receptor superfamily, member 21 | NM_014452 | 1.60 | 0.00147 |
| TNFRSF25 | tumor necrosis factor receptor superfamily, member 25 | NM_148965 | -2.69 | 3.89E-09 |
| TNFSF10 | tumor necrosis factor (ligand) superfamily, member 10 | NM_003810 | -1.67 | 5.73E-06 |
